# Examining the Relationship Between Social Support, Self-Efficacy, Diabetes Self-Management, and Quality of Life Among Rural Individuals With Type 2 Diabetes in Eastern China: Path Analytical Approach

**DOI:** 10.2196/54402

**Published:** 2024-09-19

**Authors:** Lizhu Wang, Li Li, Yang Qiu, Sihan Li, Zhonghua Wang

**Affiliations:** 1 School of Health Policy and Management Nanjing Medical University Nanjing China; 2 Binhai County People’s Hospital Yancheng China; 3 Laboratory for Digital Intelligence & Health Governance Nanjing Medical University Nanjing China

**Keywords:** Type 2 diabetes, social support, self-efficacy, self-management, quality of life, structural equation modeling

## Abstract

**Background:**

Patients with type 2 diabetes (T2D) in rural China frequently exhibit inadequate diabetes self-management (DSM) and a reduced quality of life (QoL). Social support and self-efficacy are known to influence DSM and QoL. However, the pathways through which social support and self-efficacy impact DSM and QoL among patients with T2D in rural China has yet to be fully elucidated.

**Objective:**

This study offers a foundation for developing policies in rural chronic disease management, thereby, contributing to the improvement of T2D prevention and control in China and other transitional countries.

**Methods:**

This study used a cross-sectional design, collecting data from a survey conducted between May and July 2021 on DSM and QoL among rural patients diagnosed with T2D in 2 townships in East China. All patients with T2D were enrolled through cluster sampling from the township health center database, and a questionnaire survey was administered by investigators. Structural equation modeling and multiple regression analyses were used to explore the pathways through which social support influences DSM and QoL, as well as the mediating role of self-efficacy.

**Results:**

It was found that the DSM score (mean 37.42, SD 7.70) was less than half of the maximum theoretical score. The QoL score (mean 48.92, SD 8.88) accounted for 36% of the maximum theoretical score. Social support directly and positively affected the DSM and QoL of Chinese rural patients with T2D (*P*<.01); an increase of 1 unit in social support was associated with a direct increment of 0.339 units in DSM and 0.397 units in QoL. Self-efficacy played a positive mediating role (*P*<.01), further increasing DSM and QoL by 0.147 and 0.159 units, respectively. The mediating effect of self-efficacy accounted for 30.2% and 28.6% of the total effect of social support on DSM and QoL. Furthermore, the family and friend dimension of social support, along with the symptom and disease management dimensions of self-efficacy, were significantly associated with DSM or QoL (*P*<.01).

**Conclusions:**

The study confirmed the direct and indirect influences of social support on DSM and QoL and elucidated the mediating effect of self-efficacy among rural patients with T2D in eastern China. Interventions should be developed to enhance both social support and self-efficacy, creating a positive cycle of mutual reinforcement to improve DSM and QoL among this group.

## Introduction

With an increasingly aging population, noncommunicable chronic diseases, notably diabetes mellitus, have emerged as significant global health concerns. Diabetes mellitus comprises a cluster of metabolic disorders characterized by hyperglycemia resulting from deficiencies in insulin secretion, insulin action, or both [1]. Recent data on diabetes from the 10th edition of the International Diabetes Federation’s Global Diabetes Atlas reveal that approximately 537 million individuals worldwide are living with diabetes. Notably, China harbors around 141 million patients with diabetes aged 20-79 years, constituting a quarter of the world’s diabetes patient population, with more than 90% of them diagnosed with type 2 diabetes (T2D) [2]. The diagnosed prevalence of diabetes and estimated prediabetes prevalence in mainland China stand at 12.8% and 35.2%, respectively [3]. T2D imposes a substantial burden of disease and economic losses due to its associated complications [4]. T2D significantly increases the incidence of coronary heart disease, renal failure, and the risk of diabetic foot amputation [5]. These circumstances underscore the urgent need for effective prevention, management, treatment, and financial risk mitigation strategies for patients with T2D [6].

Diabetes self-management (DSM) involves daily behaviors that individuals perform to manage their diabetes effectively. These activities include monitoring blood glucose levels, adhering to medication regimens, making dietary and exercise choices, practicing foot care, and managing treatment plans. The primary objectives are to monitor disease symptoms, enhance overall health, and mitigate the detrimental effects of diabetes on physical well-being [7]. Acknowledging the effectiveness of self-management behaviors in diabetes management, the World Health Organization has emphasized their significance over alternative interventions [8]. However, various international studies have highlighted the suboptimal DSM levels among patients with T2D across multiple countries [9-11]. This issue is particularly pronounced in rural China. Chinese rural patients with T2D tend to have lower health literacy, poorer living environments, and lower quality of health care due to the considerable disparity in urban-rural development [12-15]. Consequently, these factors contribute to suboptimal DSM level among this specific population. Quality of life (QoL) is horizontally multidimensional and comprehensive. It encapsulates an individual’s perception of their life within the framework of their cultural and value system, encompasses contextual elements such as culture, social milieu, and value systems. QoL reflects indicators related to both somatic and psychological aspects [16]. Currently, some international studies have also indicated a poorer QoL among rural patients with diabetes [17,18].

Social support is typically defined as “perceived and received social resources or assistance” [19]. It assumes a pivotal role in facilitating health-related behaviors, promoting longevity, and diminishing mortality risks [20]. Existing research suggests that social support is positively associated with engagement in self-management behaviors [21]. While health care professionals contribute timely social support through medical care, the acceptance and emotional succor from family and friends also improve patients’ self-management by shaping their perceptions of the disease [22]. Concurrently, social and family support also serve as foundational elements influencing the QoL of patients with T2D. The practical results of receiving social support and the feelings associated with it contribute to the patient’s health and emotional balance [23]. Self-efficacy denotes one’s belief in the capacity to perform specific behaviors. The literature indicates that diabetes patients with lower self-efficacy often display deficient glycemic monitoring practices [24], which is associated with low self-confidence and poor adherence to self-management routines [25]. A high level of self-efficacy can improve an individual's ability to control their environment, enhance self-management, increase confidence in facing illness, and promote physical and mental well-being, thereby helping to prevent or slow disease progression among patients with diabetes [26].

While social support emanates from external factors, self-efficacy is an inherent component of the individual. Several studies have confirmed that social support can bolster self-efficacy [27]. Patients with T2D experience a more pronounced sense of respect through larger, denser social support networks, a prerequisite for improving self-efficacy. Consequently, denser social support corresponds to enhanced self-efficacy [28,29]. As shown in the literature review mentioned above, many studies have suggested that social support and self-efficacy directly influence DSM and QoL among patients with T2D. Social support can improve self-efficacy indicating that self-efficacy may have a mediating effect between social support and DSM or social support and QoL. However, existing research highlights the correlation between social support, self-efficacy, and DSM or QoL, but few studies have examined the mediating effect of self-efficacy. Therefore, further investigation into how social support impacts DSM and QoL through the mechanism of self-efficacy is warranted.

Due to substantial disparities between urban and rural areas in China, patients with T2D from rural regions often exhibit lower levels of health literacy, experience less favorable living conditions, and receive lower-quality health care, which may result in poorer DSM and QoL. Social support and self-efficacy can directly influence DSM and QoL. However, the mediating effect of self-efficacy between social support and DSM or between social support and QoL among Chinese rural patients with T2D remains unclear. This gap in research warrants further investigation. Based on a survey of DSM and QoL among rural patients with T2D in eastern China, we conducted a path analysis to explore the direct and indirect influences of social support on DSM and QoL, to investigate the mediating effect of self-efficacy. First, we used descriptive statistics to analyze the data on social support, self-efficacy, DSM, and QoL among patients with T2D. Subsequently, we used structural equation modeling (SEM) to conduct a path analysis to uncover both the direct effect of social support and the mediating role of self-efficacy on DSM and QoL. In addition, we used multiple regression to investigate the relationships between specific dimensions of social support and self-efficacy with DSM and QoL. Consequently, this study offers a foundation for developing policies in rural chronic disease management, thereby contributing to the improvement of T2D prevention and control in China and other transitional countries.

## Methods

### Study Sample

This study used a cross-sectional design. Data were obtained from a survey conducted between May and July 2021 on DSM and QoL among rural patients diagnosed with T2D in Binhai County, Jiangsu Province, eastern China. We randomly selected 2 townships, Caiqiao and Zhenghong, from the 11 townships in Binhai County. All patients with T2D were enrolled through cluster sampling from the township health center database. A survey was then conducted using questionnaires distributed on-site by the investigators and family doctors. Respondents were rewarded with gifts upon completion. Inclusion criteria for participants encompassed the following demographic characteristics: rural residential registration; the age of 18 years or older; and confirmed T2D diagnosis as per medical records. Exclusion criteria comprised adults with cognitive impairment and mental disorders affecting communication (eg, aphasia or deafness). The questionnaire included items on patient demographics and socioeconomic factors (eg, gender, age, education, annual income, and health profiles), health management practices, social support, self-efficacy, DSM, and QoL.

A total of 2193 questionnaires were completed. Quartile-based methods were used to identify and remove outliers from the data set. Mean interpolation was applied to address missing values. Data desensitization measures were also implemented to protect patient privacy and ensure ethical compliance. As a result, we obtained 1758 valid data sets, representing an effective survey return rate of 80.16%.

### Ethical Considerations

This study was approved by the Academic Research Ethics Committee of Nanjing Medical University (reference 2021460). All procedures were in accordance with the ethical standards of the Declaration of Helsinki. Participants provided informed consent prior to data collection. Written informed consent was obtained from the individual(s) for the publication of any potentially identifiable images or data included in this article.

### Variables

#### Social Support

The Chronic Illness Resources Survey (CIRS) was used to measure patients’ social support. We used the CIRS to measure social support across 3 dimensions: health care team, family and friends, and neighborhood community. Responses were scored on a 5-point scale ranging from “none of the time” to “all of the time.” Higher scores indicated greater representations of social resources. The CIRS has been used in studies on diabetes and hypertension in China, demonstrating good validity and reliability [30,31].

#### Self-Efficacy

The self-efficacy for managing chronic disease (SECD6) scale was used to measure patients’ confidence in their abilities related to chronic disease management. It includes symptom management self-efficacy and disease management self-efficacy. Symptom management self-efficacy denotes the level of confidence in effectively managing various symptoms. Disease management self-efficacy pertains to the assurance in controlling aspects such as adhering to prescribed medications and adopting health-promoting behaviors [32]. Each item score from 1 to 10, ranging from “no confidence” to “very confident.” Higher average scores indicated a stronger sense of self-efficacy in managing chronic disease. The SECD6 has been extensively used in previous studies involving patients with various chronic diseases in China [33,34].

#### Self-Management

The Summary of Diabetes Self-Care Activities Scale (SDSCA) was used to measure DSM. It includes 10 questions that assessed 5 domains: eating plan, physical activity, blood glucose monitoring, foot care, and medication adherence. Responses were rated on a 7-point scale, ranging from “did not perform the relevant action at all during the week” to “performed it every day of the week.” Higher average scores indicated better DSM. The SDSCA has demonstrated robust reliability and validity worldwide and has been frequently used to measure DSM among patients with T2D in China [35,36].

#### Quality of Life

The diabetes-specific QoL scale (DSQL) was used to measure patients’ QoL. It includes 27 questions that assessed 4 dimensions: physiological function, psychological function, social relations, and therapeutic factors. Responses were scored using a 5-point scale, ranging from “none of the time” to “all of the time.” Higher scores indicated a greater impact of the disease and poorer QoL. The DSQL has demonstrated excellent psychometric properties and is widely used by researchers to measure the QoL of patients with T2D in China [37,38].

### Statistical Analyses

The analyses in this study were conducted in three stages: (1) descriptive statistics; (2) SEM, which includes factor analysis and path analysis to examine the influence of multiple latent variables on each other; and (3) multiple regression analysis. Specifically, we used descriptive statistics to obtain a preliminary overview of the demographic characteristics, social support, self-efficacy, DSM, and QoL among rural patients with T2D. Furthermore, we used SEM to evaluate the influence of social support on DSM and QoL, as well as the mediating effects of self-efficacy. SEM allows researchers to explore complex relationships between variables within a theoretical model by examining multiple dependencies and latent variables simultaneously [39]. In our study, social support may encourage patients to monitor blood glucose, adjust diets and lifestyles, and properly use medications, thereby improving DSM and QoL. Additionally, social support may also enhance patients’ sense of control and security, boosting their self-efficacy, which in turn improves DSM and QoL. The SEM was designed based on the variance or covariance matrix and refined by integrating measurement invariance (MI) metrics. The fitness of the trimmed path model was evaluated by calculating several fit indices. Then, a bootstrap resampling method was used to obtain accurate and stable estimates of the standard errors. In the path analysis, the direct and indirect effects of social support and self-efficacy were analyzed through maximum-likelihood covariance estimation and reported by standardized regression coefficients and *P* values. Finally, we conducted multiple regression to clarify the relationships between specific dimensions of social support, self-efficacy, and demographic variables with DSM and QoL. All analyses were conducted using SPSS (version 25.0; IBM Corp) and AMOS (version 24.0.0; IBM Corp).

## Results

### Characteristics and Descriptive Statistics of the Variables

As shown in Table 1, participants displayed a mean age of 67.69 (8.74) years, encompassing an age range spanning from 35 to 89 years. Most participants identified as female (63.94%), being unemployed (61.8%). Furthermore, a significant portion of the sample had an educational attainment below secondary school level (91.9%), were married (98.9%), and reported an annual income of less than US $1404.5 (89.5%). Nearly all participants indicated possession of medical insurance (98.9%), enrollment in family doctor services (99.8%), and maintenance of health profiles (99.8%). The results demonstrated significant differences in DSM and QoL among patients with different ages, genders, occupations, education levels, annual incomes, and health profiles. Male patients exhibited significantly higher QoL than their female counterparts (*P*<.01). In addition, patients under 65 years of age, those who are unemployed, those with a high school or higher education level, and those with higher annual incomes had better DSM and QoL compared to other patients (*P*<.05). Notably, patients participating in family doctor services exhibited better DSM scores than their nonparticipating counterparts (*P*<.01). Additionally, patients with established health profiles demonstrated enhanced DSM and QoL compared to those without such profiles (*P*<.01).

**Table 1:** Demographic characteristics, mean, and standard deviation of SDSCA^a^ and DSQL^b^.

Category		Value range	Score (minimum)	Score (maximum)	Score, mean (SD)
**Social support (CIRS^a^)**		0-5	1.67	5.00	3.70 (0.50)
	Physician and health team	0-5	1.25	5.00	4.25 (0.55)
	Family and friends	0-5	1.40	5.00	3.77 (0.65)
	Neighborhood or community	0-5	1.00	5.00	2.84 (0.93)
**Self-efficacy (SECD6^b^)**		1-10	1.67	10.00	6.36 (1.32)
	Symptom management self-efficacy	1-10	1.75	10.00	6.36 (1.32)
	Disease commonality management self-efficacy	1-10	1.00	10.00	6.37 (1.42)
**Self-management (SDSCA^c^)**		0-84	11.10	65.10	37.42 (7.70)
	Eating plan	0-7	0.00	7.00	3.74 (0.97)
	Physical activity	0-7	0.00	7.00	2.36 (1.88)
	Blood glucose monitoring	0-7	0.00	7.00	1.23 (1.06)
	Foot care	0-7	0.00	7.00	1.67 (2.18)
	Medications adherence	0-7	0.00	7.00	5.92 (2.21)
**Quality of life (DSQL^d^)**		27-135	28.00	84.00	48.92 (8.88)
	Physiological function	1-5	1.00	4.00	2.03 (0.50)
	Psychological function	1-5	1.00	3.25	1.79 (0.36)
	Social relations	1-5	1.00	3.00	1.33 (0.27)
	Therapeutic dimension	1-5	1.00	4.00	1.63 (0.40)

^a^SDSCA: Summary of Diabetes Self-Care Activities Scale.

^b^DSQL: diabetes-specific QoL scale.

### Scores for Each Dimension of Social Support, Self-Efficacy, DSM, and QoL

As shown in Table 2, the mean score of the CIRS was 3.70 (SD 0.50), with the highest score in the physician and health team dimension (mean 4.25, SD 0.55). Notably, the midpoint of the 5-point CIRS scale is 3, indicating that rural patients with T2D generally used social support to a favorable extent. The mean score of the SECD6 was 6.36 (SD 1.32). Additionally, the mean score of the SDSCA was 37.42 (SD 7.70). Specifically, medication adherence received the highest score (mean 5.92, SD 2.21), followed by eating plan (mean 3.74, SD 0.97), physical activity (mean 2.36, SD 1.88), foot care (mean 1.67, SD 2.18), and blood glucose monitoring (mean 1.23, SD 1.06). As for DSQL, the overall score was 48.92 (SD 8.88). The physiological and psychological function dimensions garnered the highest and second-highest mean scores (mean 2.03, SD 0.50; mean 1.79, SD 0.36) whereas social relations scored the lowest (mean 1.33, SD 0.27).

**Table 2:** Score details for social support, self-efficacy, diabetes self-management, and quality of life.

Category		Value range	Score (minimum)	Score (maximum)	Score, mean (SD)
**Social support (CIRS^a^)**		0-5	1.67	5.00	3.70 (0.50)
	Physician and health team	0-5	1.25	5.00	4.25 (0.55)
	Family and friends	0-5	1.40	5.00	3.77 (0.65)
	Neighborhood or community	0-5	1.00	5.00	2.84 (0.93)
**Self-efficacy (SECD6^b^)**		1-10	1.67	10.00	6.36 (1.32)
	Symptom management self-efficacy	1-10	1.75	10.00	6.36 (1.32)
	Disease commonality management self-efficacy	1-10	1.00	10.00	6.37 (1.42)
**Self-management (SDSCA^c^)**		0-84	11.10	65.10	37.42 (7.70)
	Eating plan	0-7	0.00	7.00	3.74 (0.97)
	Physical activity	0-7	0.00	7.00	2.36 (1.88)
	Blood glucose monitoring	0-7	0.00	7.00	1.23 (1.06)
	Foot care	0-7	0.00	7.00	1.67 (2.18)
	Medications adherence	0-7	0.00	7.00	5.92 (2.21)
**Quality of life (DSQL^d^)**		27-135	28.00	84.00	48.92 (8.88)
	Physiological function	1-5	1.00	4.00	2.03 (0.50)
	Psychological function	1-5	1.00	3.25	1.79 (0.36)
	Social relations	1-5	1.00	3.00	1.33 (0.27)
	Therapeutic dimension	1-5	1.00	4.00	1.63 (0.40)

^a^CIRS: Chronic Illness Resources Survey.

^b^SECD6: self-efficacy for managing chronic disease.

^c^SDSCA: Summary of Diabetes Self-Care Activities Scale.

^e^DSQL: diabetes-specific quality of life scale.

### SEM and Pathway Analysis

Firstly, we conducted a correlation analysis between social support, self-efficacy, DSM, and QoL to establish a solid foundation for the subsequent SEM and pathway analysis (Table 3). The correlation matrix was calculated to test the significance of the 2 pathways successfully: social support-self-efficacy-DSM and social support-self-efficacy-QoL. A significant and positive correlation emerged between social support and both self-efficacy and DSM (coefficient 0.247, *P*<.01; coefficient 0.124, *P*<.01). Moreover, a significant and positive correlation was discerned between self-efficacy and DSM (coefficient 0.114). Social support and self-efficacy displayed negative correlations with DSQL (coefficient –0.293, *P*<.01; coefficient –0.412, *P*<.01). However, the relationship between self-management and DSQL did not achieve statistical significance (*P*>.05).

**Table 3:** Correlation matrix and distribution of study variables.

		Social support (CIRS^a^)	Self-efficacy (SECD6^b^)	Self-management (SDSCA^c^)	QoL^d^ (DSQL^e^)
**Social support (CIRS)**					
	*r*	1			
	*P* value	—^f^			
**Self-efficacy (SECD6)**					
	*r*	0.247	1		
	*P* value	<.01	—		
**Self-management (SDSCA)**					
	*r*	0.124	0.114	1	
	*P* value	<.01	<.01	—	
**QoL (DSQL)**					
	*r*	–0.293**	–0.412**	0.004	1
	*P* value	<.01	<.01	.06	—

^a^CIRS: Chronic Illness Resources Survey.

^b^SECD6: self-efficacy for managing chronic disease.

^c^SDSCA: Summary of Diabetes Self-Care Activities Scale.

^d^QoL: quality of life.

^e^DSQL: diabetes-specific QoL scale.

^f^Not applicable.

We then constructed an original SEM to encompass social support, self-efficacy, DSM, and QoL. The adequacy of the model fit was evaluated using several fit indices, including the chi-square test (CMIN), goodness-of-fit index (GFI), root-mean-square error of approximation (RMSEA), comparative fit index (CFI), and normed fit index (NFI). Table 4 shows that the original model fit was deemed unsatisfactory compared with the good model fit, as evidenced by CMIN=20.476, GFI=0.843, RMSEA=0.101, CFI=0.843, and NFI=0.837. Therefore, the model was refined by integrating MI and aligning them with the research objectives. This recalibration led to ideal fit indices, as evidenced by CMIN=2.962, GFI=0.983, RMSEA=0.032, CFI=0.988, and NFI=0.982, thus, establishing an effective relationships model for indicators of self-efficacy, DSM, and DSQL.

**Table 4:** The fitness of indicates in structural equation modeling.

Fitness of indicates	Chi-square/*df*	GFI^a^	RMSEA^b^	CFI^c^	NFI^d^
The good model	<3	>0.90	<0.08	>0.90	>0.90
The initial model	20.476	0.843	0.101	0.843	0.837
The trimmed model	2.962	0.983	0.032	0.988	0.982

^a^GFI: goodness-of-fit index.

^b^RMSEA: root-mean-square error of approximation.

^c^CFI: comparative fit index.

^d^NFI: normed fit index.

**Figure 1:**
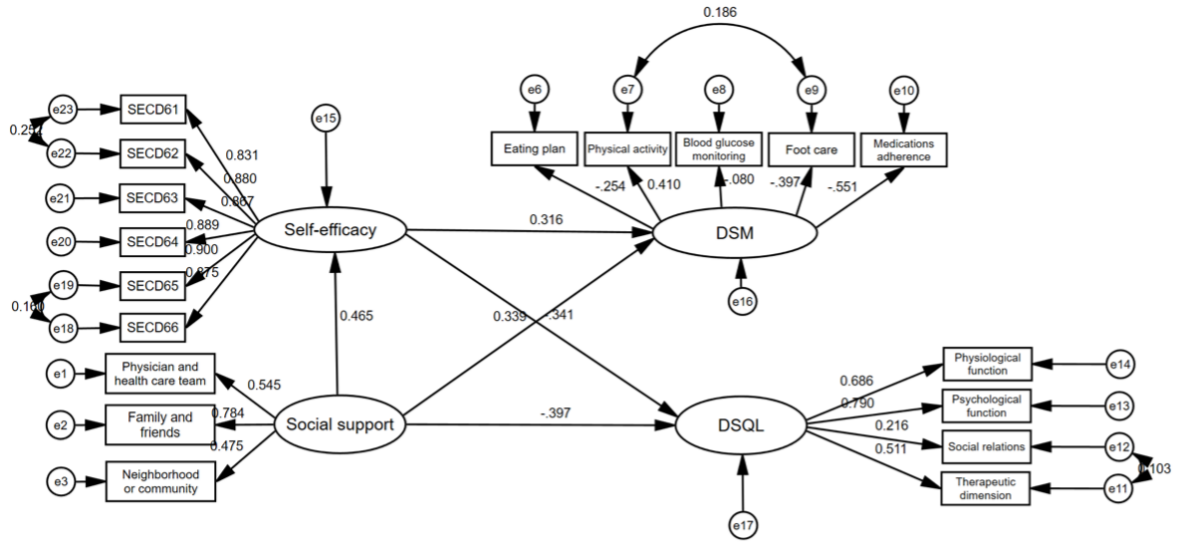
SEM for mediation by self-efficacy in the relationship among social support, DSM, and QoL.

As depicted in Figure 1 and Table 5, pathways depicting the impact of social support on DSM by bootstrap were as follows:

1. A direct and positive effect of social support on DSM (b=0.339, *P*<.001). This implies that an increase of 1 unit in social support was associated with a direct increment of 0.339 units in DSM, all other factors were constant.
2. An indirect positive effect of social support on DSM mediated through self-efficacy. The direct impact of social support on self-efficacy was 0.465 (*P*<.001), denoting that an increase of 1 unit in social support led to a corresponding increase of 0.465 units in self-efficacy, while other factors were constant. Moreover, the direct influence of self-efficacy on DSM was 0.316 (*P*<.001). Consequently, the indirect effect of social support on DSM was 0.465 × 0.316=0.147, implying that self-efficacy, operating as a mediating factor, augmented DSM by 0.147 units. The total effect of social support on DSM was 0.339 + 0.147=0.486.

**Table 5:** Test of direct effect, indirect effect, and total effect in structural equation modeling.

Endogenous variable (predicting variable)		Standardized direct effect		Standardized indirect effect		Standardized total effect	
		*β*	*P* value	*Β*	*P* value	*β*	*P* value
Self-efficacy (social support)		0.465	<.001	—^a^	—	0.465	<.001
**Diabetes self-management**							
	Social support	0.339	<.001	0.147	<.001	0.486	<.001
	Self-efficacy	0.316	<.001	—	—	0.316	<.001
**Diabetes-specific quality-of-life scale**							
	Social support	–0.397	<.001	–0.159	<.001	–0.556	<.001
	Self-efficacy	–0.341	<.001	—	—	–0.341	<.001

^a^Not applicable.

The pathways concerning social support’s influence on QoL were elucidated by bootstrap test, as follows:

1. A direct positive impact of social support on QoL (b –0.397,*P*<.001). Given that higher DSQL scores indicate diminished QoL, the coefficient signified that an increase of 1 unit in social support was associated with a direct increment of 0.397 units in QoL, whereas all other factors were constant.
2. An indirect positive effect of social support on QoL mediated through self-efficacy. The direct impact of self-efficacy on DSQL was –0.341 (*P*<.001), implying that a 1 unit rise in self-efficacy was associated with a direct increase of 0.341 units in QoL, with all other factors remaining constant. Consequently, the indirect positive effect of social support on QoL was 0.341 × 0.465=0.159, implying that self-efficacy, functioning as a mediating factor, was capable of elevating QoL by 0.159 units. The total effect of social support on QoL was 0.397 + 0.159=0.556. These results align with the results drawn from the correlation matrix and distribution.

### Multiple Regression Analysis

Table 6 presents the associations between specific dimensions of social support and self-efficacy with DSM and QOL. It was found that social support from family and friends (coefficient 0.144, *P*<.001) and neighborhood or community (coefficient 0.360, *P*<.001) were significantly and positively correlated with DSM. This effect was estimated to be greater for neighborhood or community resources. However, social support from physician and health team was negatively associated with DSM (coefficient –0.321, *P*<.001). A significant negative correlation was observed between social support from neighborhood or community (coefficient –0.181, *P*<.001) and DSQL. In addition, symptom management self-efficacy (coefficient –0.121, *P*<.001) and disease commonality management self-efficacy (coefficient –0.395, *P*<.001) had a significant negative correlation with DSQL.

**Table 6:** Association among specific dimensions of social support and self-efficacy on DSM^a^ and QoL^b^.

Variable			DSM (SDSCA^c^)					QoL (DSQL^d^)			
			*Β*	*T*		*P* value	VIF^e^	*Β*	*T*	*P* value	VIF
Constant				26.871		<.001			41.092	<.001	
**Independent variables**											
	**Social support**										
		Physician and health team	–0.321		–14.090	<.001	1.165	0.035	1.766	.08	1.165
		Family and friends	0.144		6.162	<.001	1.218	0.110	5.359	<.001	1.218
		Neighborhood or community	0.360		15.478	<.001	1.216	–0.181	–8.820	<.001	1.216
	**Self-efficacy**										
		Symptom management self-efficacy	–0.011		–0.242	.81	4.408	–0.121	–3.109	.002	4.408
		Disease commonality management self-efficacy	–0.084		–1.882	.06	4.460	–.395	–10.070	<.001	4.460
**Control variables**											
	**Gender**										
		Male	0^f^		—	—	—	0	—	—	—
		Female	–0.032		–1.436	.15	1.118	0.057	2.926	.003	1.118
	**Age**										
		<65	0		—	—	—	0	—	—	—
		≥65	–0.022		–0.995	.32	1.094	0.083	4.272	<.001	1.094
	**Occupation**										
		Unemployed	0		—	—	—	0	—	—	—
		Employed	0.004		0.166	.87	1.323	0.146	6.848	<.001	1.323
		Retired	–0.012		–0.532	.60	1.235	0.021	1.007	.31	1.235
	**Education**										
		Secondary school or below	0		—	—	—	0	—	—	—
		High school or higher	0.011		0.469	.64	1.153	–0.040	–1.988	.047	1.153
	**Marital status**										
		Married	0		—	—	—	0	—	—	—
		Unmarried	–0.028		–1.288	.20	1.048	–0.004	–0.217	.83	1.048
	**Annual income (US $)**										
		<1404.5	0		—	—	—	0	—	—	—
		1404.5-7022.6	0.037		1.555	.12	1.249	–0.072	–3.472	.001	1.249
		>7022.6	0.106		4.441	<.001	1.276	–0.026	–1.260	.21	1.276
	**Medical insurance**										
		Yes	0		—	—	—	0	—	—	—
		None	–0.002		–0.107	.92	1.062	–0.020	–1.055	.29	1.062
	**Family doctor contracting**										
		Yes	0		—	—	—	0	—	—	—
		No	–0.038		–1.784	.08	1.022	–0.003	–0.156	.88	1.022
	**Health profiles**										
		Yes	0		—	—	—	0	—	—	—
		No	–0.033		–1.568	.12	1.013	0.057	3.041	.002	1.013

^a^DSM: diabetes self-management.

^b^QoL: quality of life.

^c^SDSCA: Summary of Diabetes Self-Care Activities.

^d^DSQL: diabetes-specific quality of life.

^e^VIF: variance inflation factor.

^f^Reference values.

## Discussion

### Principal Findings

Levels of DSM and QoL among the patients with T2D have been observed to be relatively low worldwide. This holds particularly true for rural areas in China. The impact of social support on self-efficacy, DSM, and QoL among the patients with T2D has garnered significant recognition. However, few studies have delved into the mediating role of self-efficacy in the relationship between social support, DSM, and QoL. In the present study, we conducted a path analysis to explore the direct and indirect influences of social support on DSM and QoL and to investigate the mediating effect of self-efficacy among Chinese rural patients with T2D.

The results of this study revealed that younger patients with higher educational attainment and higher economic income demonstrated higher levels of DSM and QoL. Patients who are not employed exhibited higher levels of DSM and QoL. These are consistent with prior research [30,40,41]. Patients with higher literacy, better family income, or more disposable time are likely to have better resources, greater access to health knowledge, and an improved ability to understand health information, leading to better DSM and QoL. Finally, DSM scores were higher in patients who engaged with the family doctor. Family doctors provide patients with comprehensive and continuous care, ensuring regular monitoring, timely interventions to improve patients’ DSM.

The social support score was 3.70 (SD 0.50), which is higher than the urban patients with T2D, as per the literature [40,42]. This disparity may be attributed to the fact that rural communities in China often have closer-knit social networks and stronger community ties than communities in urban areas. The dimension of physician and health team support demonstrated the highest score in our study. A possible explanation is that health care professionals in rural China have developed robust community connections through the family doctor system, enabling them to provide enhanced social support for patients with T2D. The self-efficacy score was 6.36 (SD 1.32), which is lower in comparison with urban patients with T2D, as per the literature [41]. Low levels of education and limited access to information in rural China may potentially diminish the confidence of the patients with T2D in effectively managing their condition, subsequently resulting in lower self-efficacy.

The SDSCA score was 37.42 (SD 7.70), representing less than half of the maximum possible score. Among the dimensions, medication adherence behaviors scored the highest, in contrast to the results of the Iranian study on medication adherence in patients with T2D [43], whereas blood glucose monitoring behaviors scored the lowest. Rural family doctors in China frequently offer patients with T2D more medication guidance, thereby improving adherence to medication regimens. The limited emphasis on blood glucose monitoring could stem from inadequate awareness among rural patients with T2D or financial constraints restricting access to essential monitoring supplies. Therefore, strategies should focus on implementing targeted education and financial support for monitoring supplies to improve blood glucose monitoring behaviors among rural patients with T2D, ultimately leading to improved health outcomes and QoL. The DSQL score was 48.92 (SD 8.88), representing 36% of the theoretical maximum. Social relationships were the least affected dimension, suggesting that T2D has relatively minimal impact on this aspect of patients’ lives. This may be attributed to the decrease in stigma and misconceptions surrounding the disease, coupled with a notable increase in diabetes understanding within Chinese society. However, scores related to psychological and physiological functioning were relatively higher, indicating that T2D exerts a greater impact on the physical and mental health of rural patients.

The SEM elucidated that social support directly affected DSM and QoL of the rural patients with T2D, with an indirect influence through self-efficacy. In line with earlier research [44], social support played a directly pivotal role in enhancing DSM; an increase of 1 unit in social support was associated with a direct increment of 0.339 units in DSM. Social support provides individuals with emotional or practical assistance, which can enhance individuals’ understanding of their condition and improve their DSM behaviors. Furthermore, social support networks offer opportunities for individuals to share information, experiences, and knowledge related to disease management, which may provide valuable insights and strategies for effective DSM. In addition, we revealed an indirect positive effect of social support on DSM mediated through self-efficacy among rural patients with T2D. Self-efficacy, operating as a mediating factor, further augmented DSM by 0.147 units. The mediating effect of self-efficacy accounted for 30.2% of the total effects of social support on DSM. When patients receive social support in the form of encouragement, advice, and assistance, it can enhance their perception of their capabilities, leading to increased self-efficacy. Higher levels of self-efficacy, in turn, positively influence DSM behaviors. Thus, individuals who have greater confidence in their ability to manage their condition are more likely to engage in DSM. Therefore, policy efforts should focus on enhancing social support and self-efficacy through community-based programs. This includes strengthening social networks, providing training for patients and families, and developing interventions to boost self-efficacy. Continuous support from health care providers should also be ensured to address both the medical and psychosocial aspects of T2D, ultimately improving DSM for rural patients.

The study also underscored that social support significantly and directly influences the QoL of the rural patients with T2D and has a mediating effect through self-efficacy. The path coefficient emphasized the considerable direct positive effect of social support on QoL; an increase of 1 unit in social support was associated with a direct increment of 0.397 units in QoL. Support from family and friends, communities, and health professionals may have directly beneficial effects on the physical and mental health of the rural patients with T2D, thus contributing to an improved QoL for rural patients with T2D. In addition, the positive mediating role of self-efficacy was pronounced, further increasing QoL by 0.159 units. The indirect effects resulting from self-efficacy accounted for 28.6% of the total effects of social support on QoL. Social support positively impacted the self-efficacy of rural patients with T2D. Increased self-efficacy may empower patients to make healthier lifestyle choices and effectively cope with challenges, thereby enhancing their overall QoL. The mediating role of self-efficacy can facilitate the development of interventions for fostering social support and strengthening patients' self-efficacy beliefs thus creating a positive cycle of mutual reinforcement to improve QoL among rural patients with T2D.

With regard to the relationships between specific dimensions of social support and self-efficacy with DSM and QoL, our findings indicated that social support from family and friends positively influenced DSM in rural patients with T2D. This effect could be attributed to the monitoring of patient behaviors by their family and friends. Social support from neighborhood or community also exhibited a notable and positive correlation with DSM and QoL; this is similar to the results of other studies that people with high levels of community cohesion are more likely to exhibit an active lifestyle and a better QoL [45]. In rural communities of China, dense, family-centric settlements prevail, fostering intimate bonds among households and closely interconnected social interactions among community residents. The results also suggested that self-efficacy in symptom management and disease coping was associated with improving the QoL of rural patients with T2D. This contrasts with findings from a study in Tehran, which showed that dietary adherence among patients with T2D is related to their intentions [46]. Individuals demonstrating confidence in managing disease symptoms, adhering to medications, and adopting health-promoting behaviors tend to maintain optimism in confronting illness-related challenges, thereby significantly contributing to their QoL.

### Strengths and Limitation

Prior research has not examined the mediating effect of self-efficacy between social support and DSM or QoL among Chinese rural patients with T2D. Our findings confirmed the direct and indirect influences of social support on DSM and QoL, elucidated the mediating effect of self-efficacy among rural patients with T2D in eastern China. These results provide a foundation for improving chronic disease prevention and control in China and other transitional countries. However, it is crucial to acknowledge the limitations of this study. Being cross-sectional, the results of the study may be susceptible to bias. Consequently, a longitudinal study is recommended to provide deeper insights into the dynamic relationship between social support, self-efficacy, DSM, and QoL. Additionally, the study's exclusive focus on a specific rural region in eastern China may introduce sample selection bias, thereby limiting the generalizability of the findings. Finally, this study did not account for other additional factors such as health care resource and accessibility, which may influence the roles of social support and self-efficacy and impact patients' DSM and QoL. These aspects merit further investigation in future research.

### Conclusions

Our findings suggest suboptimal levels of DSM and a significant impact of T2D on QoL among Chinese rural patients with T2D. The path analysis underscored that social support directly and positively associated with the DSM and QoL of rural patients with T2D, mediated by self-efficacy. The mediating effect of self-efficacy was positive pronounced, accounting for 30.2% and 28.6% of the overall effect of social support on DSM and QoL, respectively. The dimensions of family, friends and community support and the symptom management and disease commonality management self-efficacy were significantly associated with improving DSM and QoL of patients with T2D. Hence, interventions should be developed to promote social support and self-efficacy, with particular focus on specific dimensions within each construct. This strategy aims to establish a positive cycle of mutual reinforcement, ultimately enhancing DSM and QoL among rural patients with T2D. Our findings offer a foundation for the formulation of rural chronic disease management policies, contributing to the improvement of T2D prevention and control in China and other transitional countries.

## Abbreviations

CFI: comparative fit index
CIRS: Chronic Illness Resources Survey
CMIN: chi-square test
DSM: Diabetes self-management
DSQL: Diabetes-specific quality of life
GFI: goodness-of-fit index
MI: measurement invariance
NFI: normed fit index
QoL: Quality of life
RMSEA: root-mean-square error of approximation
SDSCA: Summary of Diabetes Self-Care Activities
SECD6: Self-Efficacy for Managing Chronic Disease 6-Item
SEM: structural equation modeling
T2D: Type 2 diabetes

## Data Availability

The data sets used and analyzed during the current study are available from the corresponding author on reasonable request.
